# Performance Enhancement of INS and UWB Fusion Positioning Method Based on Two-Level Error Model

**DOI:** 10.3390/s23020557

**Published:** 2023-01-04

**Authors:** Zhonghan Li, Yongbo Zhang, Yutong Shi, Shangwu Yuan, Shihao Zhu

**Affiliations:** 1 School of Aeronautic Science and Engineering, Beihang University, Beijing 100191, China; 2 Aircraft and Propulsion Laboratory, Ningbo Institute of Technology, Beihang University, Ningbo 315100, China

**Keywords:** two-level error model, INS, UWB, DWT, EKF, fusion positioning method

## Abstract

In GNSS-denied environments, especially when losing measurement sensor data, inertial navigation system (INS) accuracy is critical to the precise positioning of vehicles, and an accurate INS error compensation model is the most effective way to improve INS accuracy. To this end, a two-level error model is proposed, which comprehensively utilizes the mechanism error model and propagation error model. Based on this model, the INS and ultra-wideband (UWB) fusion positioning method is derived relying on the extended Kalman filter (EKF) method. To further improve accuracy, the data prefiltering algorithm of the wavelet shrinkage method based on Stein’s unbiased risk estimate–Shrink (SURE-Shrink) threshold is summarized for raw inertial measurement unit (IMU) data. The experimental results show that by employing the SURE-Shrink wavelet denoising method, positioning accuracy is improved by 76.6%; by applying the two-level error model, the accuracy is further improved by 84.3%. More importantly, at the point when the vehicle motion state changes, adopting the two-level error model can provide higher computational stability and less fluctuation in trajectory curves.

## 1. Introduction

In global navigation satellite system-denied (GNSS-denied) environments, the multi-sensor fusion positioning method is mainstream, and most of them are based on INS to realize fusion positioning. With the development of micro-electro-mechanical system (MEMS) technology, applications of the inertial navigation system (INS) on small UAVs are becoming more and more extensive. This is because compared to high-precision inertial sensors, MEMS-IMU has the advantages of small size and low cost. These merits of MEMS-IMU present an attractive option for advanced applications, such as intelligent navigation and positioning in GNSS-denied environments [1]. Nevertheless, the drawbacks of high noise levels, instability of characteristics and large stochastic variance make it a challenge to use MEMS-IMU for extended periods. The noise (or error) estimation and compensation of MEMS-IMU are the key points to realize the high-precision positioning of INS-based fusion methods.

At present, INS error estimation and compensation methods are divided into two aspects: one is modeling the errors of MEMS-IMU from a mechanism level [2,3,4,5,6,7]; the other is establishing the error propagation model based on the principle of navigation. In terms of IMU error modeling methods, especially MEMS-IMU, some studies divide IMU errors into two types, which are deterministic errors, such as scale factor, bias and misalignment, and stochastic errors such as bias instability and scale factor instability [2]. The measurement of deterministic error is the main part of this type of error compensation algorithm, and extensive experimentation is required to determine the error parameters [2,3,4]. The authors of [2] summarized the methodology of how to define deterministic errors by a 27-state static test setup and a 60-state dynamic test setup and how these errors were used in error compensation models. A novel dynamic test setup was proposed in [3], which overcame the limitations in the conventional calibrations with the static or quasi-static test setups. Although an accurate error model can be established based on the above methods, many parameters need to be calibrated, and the calibration process is complicated. Other studies model inertial sensor errors from the perspective of random processes, considering MEMS-IMU errors as stochastic noise [5,8,9,10,11]. The authors of [5] proposed a model for the combined residuals and random errors, where residuals referred to the error left after the compensation of deterministic errors, and the model was still stochastic. For random noise modeling, the commonly used methods are the Allan variance [8], PSD [9], the Gauss–Markov (GM) model, the autoregressive (AR) model [10], etc. The above methods are all based on models to realize error estimation and compensation. The authors of [12,13,14,15,16] introduced unknown inputs into systems to reduce the impact of error (or noise) on the accuracy of filter estimations. Because the unknown input of the system is usually generated by factors such as environmental disturbances and mutations, it is difficult to establish a corresponding model and obtain prior information. The author of [12] derived the robust two-stage Kalman filter based on the U-V transform, which was an unknown input decoupled filter. In this method, filters were divided into bias estimator and bias-free estimator. Different from designing an unknown input decoupled filter, [13] developed the optimal filter based on the minimum variance unbiased estimation and realized the simultaneous estimation of the input and the state of a linear discrete-time system. The authors of [14] introduced an internal model approach to unknown input problems and generalized the classical internal model principle to the case with arbitrary unknown disturbances. This is a generalized model for a certain class of problems. The authors of [15] proposed a robust three-stage unscented Kalman filter and solved the unknown input problem in navigation for the Mars entry phase. The authors of [16] improved the accuracy of multiple homogeneous MEMS gyroscope fusions when suffering from unknown environmental disturbances. This article considers reducing the measurement error of IMU from the perspective of the model method and does not conduct an in-depth study of the problems of systems with unknown inputs. We summarize a set of error modeling methods. It is different from unknown input problems in that the relatively accurate prior information of noise can be obtained by using Allan variance. Additionally, the estimation errors are compensated for at the mechanism level and propagation level.

Another way to analyze and compensate for an INS error is by building an error propagation model based on navigation principles [17,18,19,20,21]. There are two basic approaches to the deviation in INS error models in the literature: the phi-angle approach (or the true frame approach) and the psi-angle approach (or the computer frame approach) [17,18]. The attitude calculation in INS has three approaches: Euler angle, direction cosine matrix and quaternion. The quaternion method has received extensive attention for the advantages of less computation, higher accuracy and avoiding singularities [20,21]. Nevertheless, most of the error propagation models were established based on the geographic frame and relied on latitude and longitude to provide location information. In GNSS-denied environments, such as an indoor environment, these error models were inconvenient to use. Meanwhile, there are a few pieces of research combining the mechanism error model with the propagation error model.

This paper aims to comprehensively utilize the mechanism error model and propagation error model, propose the two-level error model and summarize a complete set of methods from data prefiltering to data fusion. The rest of this article is organized as follows. In Section 2, we introduce the data prefiltering method in relation to raw IMU data. In Section 3, we propose the two-level error model and EKF fusion method based on this. The different experiments and results are shown in Section 4. Section 5 summarizes this article.

## 2. Data Prefiltering

The vast majority of multi-sensor positioning methods are based on INS, which rely on IMU, especially MEMS-IMU, to achieve positioning and navigation. MEMS-IMU includes one accelerometer and one gyroscope, which can measure three-axes acceleration and three-axes angular velocity. The measurements of MEMS-IMU always contain a certain amount of noise, which leads to non-negligible drifts in vehicle path estimation due to time integration. There are two categories of noise: short-term noise with a high frequency and long-term noise with a low frequency. In the time domain, the vehicle motion information mixes with such noise, and they cannot be distinguished directly. While in the frequency domain, the movement frequency of a vehicle is usually no more than 20 Hz, and a land vehicle is usually below 5 Hz [22], whereas short-term noise can reach more than 50 Hz. Therefore, we need to employ data prefiltering methods before utilizing the two-level error model to eliminate short-term noise whose frequency is around the vehicle movement frequency.

### 2.1. Wavelet Denoising Method

Fourier transform (FT) is the most common and extremely useful method for frequency analysis. However, through transforming the signal to the frequency domain by FT, the time-domain information is lost. Short-time Fourier transform (STFT) can overcome this drawback by adding a window function. However, both time localization and frequency resolution cannot be optimal at the same time. Therefore, performing a wavelet transform to realize IMU signal decomposition in both the time and frequency domains is a superior method.

From multiresolution analysis, $\phi \left(x\right)$ and $\psi \left(x\right)$ are the scaling function and wavelet function, respectively, and they satisfy the following equations:

(1) $$\phi \left(x\right)=\sqrt{2}\sum_{n}h_{n}\phi \left(2x-n\right)$$

(2) $$\psi \left(x\right)=\sqrt{2}\sum_{k}g_{k}\phi \left(2x-k\right)$$

The coefficients of the scaling and wavelet functions obey the following equations:

(3) $$h_{n}=\sqrt{2}\int \phi \left(x\right)\phi \left(2x-n\right)dx$$

(4) $$g_{k}=\sqrt{2}\int \psi \left(x\right)\phi \left(2x-k\right)dx$$

where $h_{n}$ has the characteristic of a finite impulse response (FIR) low-pass digital filter, and the FIR digital filter that consists of the wavelet coefficient, $g_{k}$, is a high-pass digital filter [23]. By performing discrete-time FT as Equations (5) and (6), we can get the frequency response of both filters.

(5) $$H\left(w\right)=\sum_{n=-\infty }^{\infty }h_{n}e^{-inw}$$

(6) $$G\left(w\right)=\sum_{k=-\infty }^{\infty }g_{k}e^{-ikw}$$

**Figure 1:**
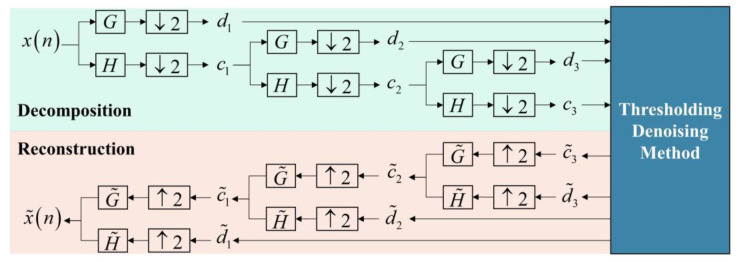
The diagram of wavelet denoising method: take three-level decomposition as an example.

Based on the Mallat algorithm [24] and the filter bank method, the wavelet denoising method is divided into three parts: decomposition, threshold denoising method and reconstruction. For example, the three-level decomposition is shown in Figure 1. $x\left(n\right)$ is the raw IMU signal in the time domain. By applying convolution with digital filter banks derived from the scaling and wavelet functions, the raw signal is decomposed into the wavelet coefficient, $d_{1}$, with high frequency and the scaling coefficient, $c_{1}$, with low frequency. Both $d_{1}$ and $c_{1}$ are in the frequency domain. After three times repetitions, there are four parts of the signal corresponding to different frequency intervals. The coefficients containing motion information have a large amplitude, so setting a suitable thresholding in specific methods can eliminate a certain amount of noise. After thresholding denoising, by implementing the inverse wavelet transform that is $\tilde{G}$ and $\tilde{H}$ in Figure 1 on ${\tilde{c}}_{3}$ and ${\tilde{d}}_{3}$, we can get the reconstructed coefficient, ${\tilde{c}}_{2}$. Additionally, if repeated three times, the denoised IMU signal, $\tilde{x}\left(n\right)$, can be obtained. In the decomposition part, digital filter banks and downsampling are taken to realize the discrete wavelet transform. While in the reconstruction part, digital filter banks and upsampling are taken to reconstruct the signal.

Hard thresholding [25] and soft thresholding [26] are two common thresholding methods, and some scholars have conducted in-depth research on the selection of thresholds [25,27,28,29]. In the hard-thresholding method, coefficients with amplitudes less than thresholding are set to 0, and coefficients that are greater than thresholding remain unchanged, and the equation is as (7).

(7) $$T^{hard}\left(x\right)=\left\{\begin{array}{c} 0,\;\left|x\right|\leq T\\ x,\;\left|x\right|>T \end{array}\right.$$

While in the soft-thresholding method, coefficients with magnitudes less than thresholding are set to 0, and coefficients with magnitudes larger than thresholding are reduced to the difference in them, and the equation is

(8) $$T^{soft}\left(x\right)=\left\{\begin{array}{cc} 0, & \left|x\right|\leq T\\ \mathrm{sgn}\left(x\right)\left(\left|x\right|-T\right), & \left|x\right|>T \end{array}\right.$$

Considering the characteristics of the raw IMU signal, this paper presents the soft-thresholding method for IMU data denoising. More detailed information will be introduced in Section 2.2.

### 2.2. Implementation Details

The fundamental theory of the wavelet shrinkage method is that wavelet function has a better time–frequency property, and discrete wavelet transform (DWT) has an ability to “focus” on signals because of its multiresolution property. The “focus” ability makes signal energy fasten on several coefficients, while noise is evenly distributed across the whole scale space, and this is determined by the distribution property of noise.

The IMU data acquired from MEMS-IMU usually include a large amount of noise; one reason is that MEMS-IMU lacks high accuracy, and the other is that the movements of a vehicle always bring vibrations, and this affects the measurement of MEMS-IMU. We perform two levels of wavelet decomposition on account of the UAV motion frequency and IMU sampling frequency. Based on the soft-thresholding method, we selected the SURE-Shrink algorithm to evaluate the noise threshold. This algorithm is a hybrid algorithm based on the SURE threshold and Universal threshold; it considers the differences in the statistical properties of the coefficients of different wavelet sub-bands, and it is one of the best sub-band adaptive wavelet shrinkage algorithms. The calculation is as follows:

(9) $$T=\left\{\begin{array}{c} T_{sure},\;S_{N}^{2}\leq \frac{\eta_{N}}{\sqrt{N}}\\ T_{univ},\;S_{N}^{2}>\frac{\eta_{N}}{\sqrt{N}} \end{array}\right.$$

where

(10) $$\eta_{N}=\log_{2}{\left(N\right)}^{3/2}$$

(11) $$S_{N}^{2}=\sum_{t}\left(Y_{t}^{2}-1\right)/N$$

Here, N is the number of coefficients of the wavelet sub-band, and $Y_{t}$ is the t-th coefficient of the current wavelet sub-band. The two thresholds, $T_{univ}$ and $T_{sure}$, are calculated by

(12) $$T_{univ}=\sigma_{n}\sqrt{2\ln N}$$

(13) $$T_{sure}=\arg\underset{T>0}{\min}\left\{N\cdot \sigma_{n}^{2}+\sum_{i=0}^{N-1}\max\left\{Y_{i},T^{2}\right\}-2\sigma_{n}^{2}\#\left\{\left|Y_{t}\right|\leq T\right\}\right\}$$

where $\#\left\{\cdot \right\}$ represents the number of elements that satisfy the conditions. From [25], $\sigma_{n}$ can be estimated as the median absolute deviation in the wavelet coefficients at the finest level and divided by 0.6745. The finest level of the wavelet coefficients contains the highest frequency level of the signal, which we think most of them are noise. Additionally, the equation form is

(14) $$\sigma_{n}=median\left(\left|d_{N}-median\left(d_{N}\right)\right|\right)/0.6745$$

**Figure 2:**
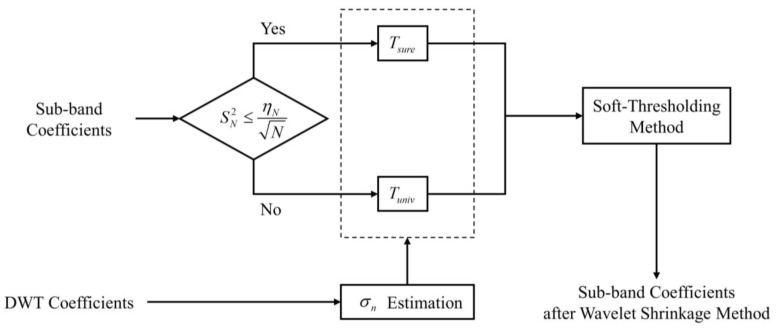
Diagram of soft-thresholding algorithm based on SURE-Shrink method.

Here, $d_{N}$ is the wavelet coefficients of the finest level. Additionally, the flow chart of the wavelet shrinkage algorithm is shown in Figure 2.

The denoising results are detailed in Section 4.1.2.

## 3. Methodology

In this chapter, according to the mechanism of MEMS-IMU errors and the motion characteristics of UAV, we introduce the two-level error model EKF method in detail, which is the main innovation of this paper. The two-level error model is established from two levels: one is from the mechanism, and the other is from the propagation. In the mechanism error model, we aim to build an error model that can describe the accelerometer and gyroscope sensor errors simply and generally. While in the propagation error model, we intend to establish the error propagation path based on the state equations of the navigation system.

### 3.1. Mechanism Error Model

From the mechanism of error occurrence, the error variables we focus on are δa and δw, which are errors of state a and w. For these two variables, we selected the stochastic process model as the mechanism error model; the arguments are as follows:

1. It is convenient to analyze and calculate the parameters of the stochastic model;
2. There are little application condition limitations on the stochastic model;
3. There is no need to calibrate a large number of parameters in contrast to other methods.

The common stochastic models used to describe errors are the autoregressive (AR) model and Gauss–Markov (GM) process model. More detailed information about the mechanism error model is discussed in Section 3.1.1 and Section 3.1.2.

#### 3.1.1. Stochastic Process Model

In most KF implementations for the INS-based fusion positioning method, the first-order GM model is used to describe inertial sensor errors with a decaying exponential autocorrelation sequence [30,31,32,33]. The first-order GM model for an inertial sensor error is given as:

(15) $$\dot{x}\left(t\right)=-\beta x\left(t\right)+\sqrt{2\beta \sigma^{2}}w\left(t\right)$$

Here, β is the reciprocal of correlation time, and $\sigma^{2}$ is the variance in system noise $w\left(t\right)$. The discrete-time equation is written as follows:

(16) $$x_{k}=\left(I-\beta \Delta t\right)x_{k-1}+\sqrt{2\beta \sigma^{2}}w_{k}\Delta t$$

where Δt is the time interval.

The other stochastic model of inertial sensor errors is the higher-order AR model. To use this model, long-term measurements from each inertial sensor while stationary are required for computing the higher-order AR model. The pth-order AR model for a discrete-time-domain sequence can be described by the following difference equation:

(17) $$y\left(n\right)=-\sum_{k=1}^{p}\alpha_{k}y\left(n-k\right)+\beta_{0}w\left(n\right)$$

where $\alpha_{1},\alpha_{2},\alpha_{3},\ldots ,\alpha_{p}$ are the model parameters, and $\beta_{0}$ is standard deviation in sensor white noise. However, to achieve higher accuracy, the model needs to use a higher order. Since the AR model is applied to all six axes of inertial sensors, each increase in the model order will lead to six more states added to the KF state error vector.

#### 3.1.2. Implementation Details

Our aim in this paper is not to establish a noise model to describe the physical properties of sensor errors in detail but merely to derive generic, simple noise models that are suitable for the INS-based fusion positioning method. The mechanism error model is derived from (16); in the discrete-time domain, the equation is as follows:

(18) $$x_{k+1}=\left(I-\frac{\Delta t}{\tau_{b}}\right)x_{k}+w_{k}+v_{k}$$

where $x_{k}$ is the slowly varying process with the correlation time, $\tau_{b}$, in the discrete-time domain, $w_{k}$ is the white noise component of $x_{k}$, and $v_{k}$ represents the white noise component of the error model. and v are independent, zero mean, white Gaussian processes of strength $\sigma_{b}$ and $\sigma_{w}$, and in the discrete-time domain:

(19) $$E\left[w_{k}w_{k}^{T}\right]=\Delta t\cdot \sigma_{b}^{2}$$

(20) $$E\left[v_{k}v_{k}^{T}\right]=\frac{\sigma_{w}^{2}}{\Delta t}$$

We define $\bar{w}=w+v$, and then

(21) $$E\left[\bar{w}{\bar{w}}^{T}\right]=\Delta t\cdot \sigma_{b}^{2}+\frac{\sigma_{w}^{2}}{\Delta t}$$

Now the mechanism error model in the discrete-time domain becomes


(22) $$x_{k+1}=\left(I-\frac{\Delta t}{\tau_{b}}\right)x_{k}+{\bar{w}}_{k}$$

When we apply the mechanism error model to six axes of inertial sensors, the parameters that need to be obtained beforehand are $\theta =\left\{\tau_{b},\sigma_{b},\sigma_{w}\right\}$. By keeping the IMU stationary for more than 3 h, which means the outputs of IMU only contain noise (including long-term noise and short-term noise), the parameters $\sigma_{b}$, $\sigma_{w}$ can be directly read off of the Allan deviation plot [33], and $\tau_{b}$ is the correlation time of the output signals.

### 3.2. Propagation Error Model

For the propagation error model, we derive model equations based on the state differential equations of navigation systems. The variables we focus on are δp, δv, δq, which are errors of state p, v, q. We use quaternions, q, instead of Euler angles to describe the rotations of navigation systems because solving in quaternions can avoid singularities and the gimbal lock problem. The differential equations that characterize the motion of navigation systems are given as:

(23) $$\dot{p}\left(t\right)=v\left(t\right)$$

(24) $$\dot{v}\left(t\right)=C\left[q\left(t\right)\right]a\left(t\right)+g\left[p\left(t\right)\right]$$

(25) $$\dot{q}\left(t\right)=\Omega \left[w\left(t\right)\right]q\left(t\right)=Q\left[q\left(t\right)\right]w\left(t\right)$$

where p,v are the position and velocity of the navigation systems, respectively, in the inertial reference frame; a is the specific acceleration vector; the gravitational acceleration $g\left[p\left(t\right)\right]$ changes with the unit position; and the rotation matrix based on Hamilton form can be calculated by

(26) $$C\left[q\left(t\right)\right]=\left[\begin{array}{ll} q_{0}^{2}+q_{1}^{2}-q_{2}^{2}-q_{3}^{2} & 2q_{1}q_{2}-2q_{0}q_{3}\\ 2q_{0}q_{3}+2q_{1}q_{2} & q_{0}^{2}-q_{1}^{2}+q_{2}^{2}-q_{3}^{2}\\ 2q_{1}q_{3}-2q_{0}q_{2} & 2q_{0}q_{1}+2q_{2}q_{3} \end{array}\right.\left.\begin{array}{c} 2q_{0}q_{2}+2q_{1}q_{3}\\ 2q_{2}q_{3}-2q_{0}q_{1}\\ q_{0}^{2}-q_{1}^{2}-q_{2}^{2}+q_{3}^{2} \end{array}\right]$$

The differential equation of quaternion is given as (27):

(27) $$\dot{q}=\frac{1}{2}q⊗w=Q(q)\cdot w=\Omega (w)\cdot q$$

where w is the angular velocity, and $q={\left[q_{w},q_{v}\right]}^{T}$ is the Hamilton form of quaternions with the scalar part, $q_{w}$, and the vector part, $q_{v}$; in this form, rotation corresponds to the right-hand rule. ⊗ denotes quaternion multiplication; the latter two are equivalent forms, which are matrix multiplication rather than quaternion multiplication. Additionally, the matrix $\Omega \left(w\right),Q\left(q\right)$ is given as:

(28) $$\Omega (w)=\frac{1}{2}\left[\begin{array}{cccc} 0 & -w_{1} & -w_{2} & -w_{3}\\ w_{1} & 0 & w_{3} & -w_{2}\\ w_{2} & -w_{3} & 0 & w_{1}\\ w_{3} & w_{2} & -w_{1} & 0 \end{array}\right]=\frac{1}{2}\left[\begin{array}{cc} 0 & -w^{T}\\ w & -{\left[w\right]}_{\times } \end{array}\right]$$

(29) $$Q\left(q\right)=\frac{1}{2}\left[\begin{array}{ccc} -q_{1} & -q_{2} & -q_{3}\\ q_{0} & -q_{3} & q_{2}\\ q_{3} & q_{0} & -q_{1}\\ -q_{2} & q_{1} & q_{0} \end{array}\right]$$

(30) $${\left[V\right]}_{\times }=\left[\begin{array}{ccc} 0 & -V_{3} & V_{2}\\ V_{3} & 0 & -V_{1}\\ -V_{2} & V_{1} & 0 \end{array}\right]$$

Equations (23)–(25) characterize the true attitude and position of a navigation system based on true specific acceleration and angular velocity. However, in an actual system, only measurements are available. For a quantity z, $z_{m}=$ the measurement of z and δz= the error of z. Additionally, the true quantity can be characterized by the sum of measurement and error. In the navigation system, there are equations listed as (31)–(35).

(31) $$p=p_{m}+\delta p$$

(32) $$v=v_{m}+\delta v$$

(33) $$q=q_{m}+\delta q$$

(34) $$a=a_{m}+\delta a$$

(35) $$w=w_{m}+\delta w$$

The differential equation of the position error can be derived as follows:

(36) $$\delta \dot{p}=\dot{p}-{\dot{p}}_{m}=v-v_{m}=\delta v$$

The differential equation of the velocity error can be derived as:

(37) $$\begin{array}{c} \delta \dot{v}=\dot{v}-{\dot{v}}_{m}=C\left(q\right)a+g\left(p\right)-C\left(q_{m}\right)a_{m}-g\left(p_{m}\right)\\ \;\;\;\;=C\left(q_{m}+\delta q\right)\left(a_{m}+\delta a\right)+g\left(p_{m}+\delta p\right)-C\left(q_{m}\right)a_{m}-g\left(p_{m}\right) \end{array}$$

The rotation matrix and gravitational acceleration can be approximated with measurements, with first-order errors, as (38) and (39).

(38) $$C\left(q_{m}+\delta q\right)=C\left(q_{m}\right)+\frac{\partial C}{\partial q_{m}}\cdot \delta q$$

(39) $$g\left(p_{m}+\delta p\right)=g\left(p_{m}\right)+\frac{\partial g}{\partial p_{m}}\cdot \delta p$$

Then, the new differential Equation (40) of the velocity error can be obtained from (38) and (39). Considering sufficiently small errors, the products of errors can be neglected, and (40) can finally become (41).

(40) $$\delta \dot{v}=\left[C\left(q_{m}\right)+\frac{\partial C}{\partial q_{m}}\cdot \delta q\right]\cdot \left(a_{m}+\delta a\right)+\left[g\left(p_{m}\right)+\frac{\partial g}{\partial p_{m}}\cdot \delta p\right]-C\left(q_{m}\right)a_{m}-g\left(p_{m}\right)$$

(41) $$\delta \dot{v}=C\left(q_{m}\right)\cdot \delta a+\frac{\partial C}{\partial q_{m}}a_{m}\cdot \delta q+\frac{\partial g}{\partial p_{m}}\cdot \delta p$$

The differential equation of quaternion can be derived as

(42) $$\begin{array}{c} \delta \dot{q}=\dot{q}-{\dot{q}}_{m}=\Omega \left(w_{m}+\delta w\right)\cdot \left(q_{m}+\delta q\right)-Q\left(q_{m}\right)\cdot w_{m}\\ \;\;\;\;=\Omega \left(w_{m}+\delta w\right)\cdot \delta q+\Omega \left(w_{m}+\delta w\right)\cdot q_{m}-Q\left(q_{m}\right)\cdot w_{m} \end{array}$$

It can be easily approved that $Q\left(q\right)\cdot w=\Omega \left(w\right)\cdot q$, and (42) becomes

(43) $$\begin{array}{c} \delta \dot{q}=\Omega \left(w_{m}+\delta w\right)\delta q+Q\left(q_{m}\right)\left(w_{m}+\delta w\right)-Q\left(q_{m}\right)w_{m}\\ \;\;\;\;=\Omega \left(w_{m}+\delta w\right)\delta q+Q\left(q_{m}\right)\delta w \end{array}$$

Neglecting the products of small errors, the error equation of quaternions becomes

(44) $$\delta \dot{q}=\Omega \left(w_{m}\right)\delta q+Q\left(q_{m}\right)\delta w$$

The propagation error model can finally be obtained:

(45) $$\delta \dot{p}=\delta v$$

(46) $$\delta \dot{v}=C\left(q_{m}\right)\cdot \delta a+\frac{\partial C}{\partial q_{m}}a_{m}\cdot \delta q+\frac{\partial g}{\partial p_{m}}\cdot \delta p$$

(47) $$\delta \dot{q}=\Omega \left(w_{m}\right)\delta q+Q\left(q_{m}\right)\delta w$$

### 3.3. Error Model EKF Method

#### 3.3.1. Basic EKF Method

The continuous-time nonlinear system’s state equation is

(48) $$\dot{x}=f\left(x,u,w,t\right),\;w\sim \left(0,Q\right)$$

Additionally, the continuous-time nonlinear measurement equation is

(49) $$y=h\left(x,v,t\right),\;v\sim \left(0,R\right)$$

The discrete-time nonlinear system equations can be obtained by discretization, and (48) and (49) become

(50) $$x_{k}=f_{k-1}\left(x_{k-1},u_{k-1},w_{k-1}\right),\;w_{k}\sim \left(0,Q_{k}\right)$$

(51) $$y_{k}=h_{k}\left(x_{k},v_{k}\right),\;v_{k}\sim \left(0,R_{k}\right)$$

Applying Taylor series expansion and keeping the first order at $x_{k-1}={\hat{x}}_{k-1}^{+}$, the state Equation (50) becomes

(52) $$x_{k}=F_{k-1}x_{k-1}+{\tilde{u}}_{k-1}+L_{k-1}w_{k-1}$$

Here,

(53) $$F_{k-1}={\left.\frac{\partial f_{k-1}}{\partial x}\right|}_{{\hat{x}}_{k-1}^{+}},L_{k-1}={\left.\frac{\partial f_{k-1}}{\partial w}\right|}_{{\hat{x}}_{k-1}^{+}}$$

(54) $${\tilde{u}}_{k-1}=f_{k-1}\left({\hat{x}}_{k-1}^{+},u_{k-1},0\right)-F_{k-1}{\hat{x}}_{k-1}^{+}$$

Additionally, the nonlinear measurement equation becomes (55) after a first-order approximation at $x_{k}={\hat{x}}_{k}^{-}$.

(55) $$y_{k}=H_{k}x_{k}+z_{k}+M_{k}v_{k}$$

Here,

(56) $$H_{k}={\left.\frac{\partial h_{k}}{\partial x}\right|}_{{\hat{x}}_{k}^{-}},M_{k}={\left.\frac{\partial h_{k}}{\partial v}\right|}_{{\hat{x}}_{k}^{-}}$$

(57) $$z_{k}=h_{k}\left({\hat{x}}_{k}^{-},0\right)-H_{k}{\hat{x}}_{k}^{-}$$

Here, the basic Kalman filter equations can be applied after the above discretization and linearization; there are the state estimations of (58) and (59) and the measurement updates of (60)–(62).

(58) $$P_{k}^{-}=F_{k-1}P_{k-1}^{+}F_{k-1}^{T}+L_{k-1}Q_{k-1}L_{k-1}^{T}$$

(59) $${\hat{x}}_{k}^{-}=f_{k-1}\left({\hat{x}}_{k-1}^{+},u_{k-1},0\right)$$

(60) $$K_{k}=P_{k}^{-}H_{k}^{T}{\left(H_{k}P_{k}^{-}H_{k}^{T}+M_{k}R_{k}M_{k}^{T}\right)}^{-1}$$

(61) $${\hat{x}}_{k}^{+}={\hat{x}}_{k}^{-}+K_{k}\left[y_{k}-h_{k}\left({\hat{x}}_{k}^{-},0\right)\right]$$

(62) $$P_{k}^{+}=\left(I-K_{k}H_{k}\right)P_{k}^{-}$$

#### 3.3.2. Two-Level Error Model EKF Method

In this section, the two-level error model is applied to the EKF method. We take the ultra-wideband (UWB) system as the measurement and derive the EKF method based on this. The state vectors that need to be considered include p: position; v: velocity; and q: quaternion; especially, in this paper, we need to discriminate their nominal states and error states. Therefore, the state vector is given as follows:

(63) $$x={\left[p^{1\times 3},v^{1\times 3},q^{1\times 4},\delta p^{1\times 3},\delta v^{1\times 3},\delta q^{1\times 4},\delta a^{1\times 3},\delta w^{1\times 3}\right]}^{T}$$

where δa is the error state of acceleration obtained by the accelerometer, and δw is the error state of angular velocity obtained by the gyroscope. The state vector, x, is a 26×1 column vector. In order to facilitate the subsequent matrix derivation, the following statements are given here. Variables except for quaternion, which is a 1×4 row vector, are composed of three elements, which are (we take the position as an example): $p^{1\times 3}=\left[p_{1},p_{2},p_{3}\right]$, and they match the x, y and z axes, respectively.

**Figure 3:**
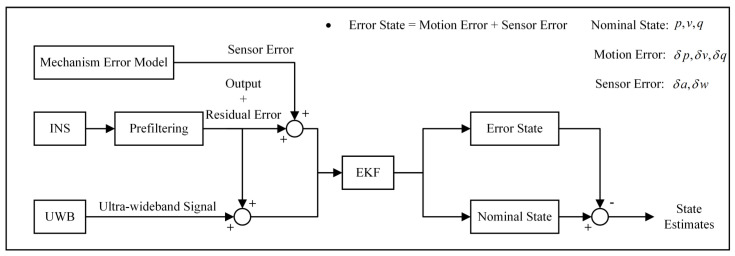
Data fusion method based on two-level error model.

The elements in the state vector are divided into three groups: sensor errors corresponding to mechanism error model variables, δa,δw; motion errors corresponding to propagation error model variables, δp,δv,δq; and motion nominal state variables, p,v,q. Modifications need to be applied to the traditional EKF method to adopt the two-level error model. At the moment, k, the state estimation of the navigation system is divided into two layers: (a) the motion nominal state performs a prediction based on sensor errors of moment k-1 and is updated based on UWB measurements at the moment, k; (b) the motion errors perform a prediction based on their state at the moment, k-1, and sensor errors at the moment, k-1. Additionally, the sensor errors at the moment, k, are predicted by the mechanism error model. The true motion state of the navigation system at the moment, k, is the sum of the motion nominal state and motion errors. The data fusion process is shown in Figure 3.

The state equations with propagation error models in continuous time are as follows:

(64) $$\dot{p}=v$$

(65) $$\dot{v}=C\cdot \left(a_{m}+\delta a\right)+g$$

(66) $$\dot{q}=\frac{1}{2}q⊗\left(w_{m}+\delta w\right)$$

(67) $$\delta \dot{p}=\delta v$$

(68) $$\delta \dot{v}=C\left(q\right)\cdot \delta a+\frac{\partial C}{\partial q}a_{m}\cdot \delta q+\frac{\partial g}{\partial p}\cdot \delta p$$

(69) $$\delta \dot{q}=\Omega \left(w_{m}\right)\delta q+Q\left(q\right)\delta w$$

By discretization, (64)–(69) become

(70) $$p^{k}=p^{k-1}+\frac{1}{2}\left(v^{k}+v^{k-1}\right)\cdot \Delta t$$

(71) $$v^{k}=v^{k-1}+\left[C^{k-1}\left(a_{m}^{k-1}+\delta a^{k-1}\right)+g^{k-1}\right]\cdot \Delta t$$

(72) $$q^{k}=q^{k-1}⊗q\left\{\left(w_{m}^{k-1}+\delta w^{k-1}\right)\cdot \Delta t\right\}$$

(73) $$\delta p^{k}=\delta p^{k-1}+\frac{1}{2}\left(\delta v^{k}+\delta v^{k-1}\right)\cdot \Delta t$$

(74) $$\delta v^{k}=\delta v^{k-1}+\left[{\left(\frac{\partial C}{\partial q}\right)}^{k-1}\cdot a_{m}^{k-1}\cdot \Delta t\right]\cdot \delta q^{k-1}+C\left(q^{k-1}\right)\cdot \delta a^{k-1}\cdot \Delta t$$

(75) $$\delta q^{k}=Q\left(q^{k-1}\right)\cdot \delta w^{k-1}+\Omega \left(w_{m}^{k-1}\right)\cdot \delta q^{k-1}$$

Additionally, the mechanism error models are

(76) $$\delta a_{k+1}=\left(I-\frac{\Delta t}{\tau_{b,a}}\right)\delta a_{k}+{\bar{w}}_{a,k}$$

(77) $$\delta w_{k+1}=\left(I-\frac{\Delta t}{\tau_{b,w}}\right)\delta w_{k}+{\bar{w}}_{w,k}$$

where

(78) $$E\left[{\bar{w}}_{a,k}{\bar{w}}_{a,k}^{T}\right]=\Delta t\cdot \sigma_{b,a}^{2}+\frac{\sigma_{w,a}^{2}}{\Delta t}$$

(79) $$E\left[{\bar{w}}_{w,k}{\bar{w}}_{w,k}^{T}\right]=\Delta t\cdot \sigma_{b,w}^{2}+\frac{\sigma_{w,w}^{2}}{\Delta t}$$

Applying Taylor series expansion and keeping the first order, the state equations finally become

(80) $$x_{k}=F\cdot x_{k-1}+G\cdot w_{k-1}$$

where $w_{k-1}={\left[w_{a}^{k-1},w_{w}^{k-1}\right]}^{T}$, and $w_{a}$ is the associated white noise process of the accelerometer with the known covariance; $w_{w}$ is that of the gyroscope with the known covariance. The state transition matrix *F* is a 26×26 matrix and is calculated as:

(81) $$F=\left[\begin{array}{llllllll} F_{11} & F_{12} & 0 & 0 & 0 & 0 & 0 & 0\\ 0 & F_{22} & F_{23} & 0 & 0 & 0 & F_{27} & 0\\ 0 & 0 & F_{33} & 0 & 0 & 0 & 0 & F_{38}\\ 0 & 0 & 0 & F_{44} & F_{45} & 0 & 0 & 0\\ 0 & 0 & 0 & 0 & F_{55} & F_{56} & F_{57} & 0\\ 0 & 0 & 0 & 0 & 0 & F_{66} & 0 & F_{68}\\ 0 & 0 & 0 & 0 & 0 & 0 & F_{77} & 0\\ 0 & 0 & 0 & 0 & 0 & 0 & 0 & F_{88} \end{array}\right]$$

The derivations in the individual elements of the state transition matrix *F* can be found in Appendix A. For the noise vectors $w_{a}$ and $w_{w}$, the noise coefficient matrix *G* is a 26×6 matrix and is calculated by

(82) $$G={\left[\begin{array}{llllllll} 0 & 0 & 0 & 0 & 0 & 0 & I_{3\times 3}\cdot \Delta t & 0\\ 0 & 0 & 0 & 0 & 0 & 0 & 0 & I_{3\times 3}\cdot \Delta t \end{array}\right]}^{T}$$

The measurement vector z contains four distances because of the deployment of four UWB base stations. The distance is measured based on the arrival time difference of electromagnetic waves. Additionally, the vector z is given as (83).

(83) $$z={\left[d_{1},d_{2},d_{3},d_{4}\right]}^{T}$$

(84) $$d_{i}\left(k\right)=\sqrt{{\left(p_{1}\left(k\right)-x_{i}\right)}^{2}+{\left(p_{2}\left(k\right)-y_{i}\right)}^{2}+{\left(p_{3}\left(k\right)-z_{i}\right)}^{2}},\;i=1,2,3,4$$

where $p\left(k\right)={\left[p_{1}\left(k\right),p_{2}\left(k\right),p_{3}\left(k\right)\right]}^{T}$, in which subscript (1,2,3) corresponds with the axes $\left(x,y,z\right)$. $\left(x_{i},y_{i},z_{i}\right)$ make up the coordinate of the i-th UWB base station.

The discrete-time measurement equation is given by (85):

(85) z=H·x+r

where *H* is the measurement coefficient matrix, and r is the measurement noise vector, which is the assumed white noise. The matrix *H* is derived by (86).

(86) $$H_{4\times 26}=\left[H_{p,4\times 3}^{d},0_{4\times 23}\right]$$

The matrix $H_{p,4\times 3}^{d}$ has the form of

(87) $$H_{p,4\times 3}^{d}=\left[\begin{array}{lll} \frac{p_{1}\left(k\right)-x_{1}}{R_{1}} & \frac{p_{2}\left(k\right)-y_{1}}{R_{1}} & \frac{p_{3}\left(k\right)-z_{1}}{R_{1}}\\ \frac{p_{1}\left(k\right)-x_{2}}{R_{2}} & \frac{p_{2}\left(k\right)-y_{2}}{R_{2}} & \frac{p_{3}\left(k\right)-z_{2}}{R_{2}}\\ \frac{p_{1}\left(k\right)-x_{3}}{R_{3}} & \frac{p_{2}\left(k\right)-y_{3}}{R_{3}} & \frac{p_{3}\left(k\right)-z_{3}}{R_{3}}\\ \frac{p_{1}\left(k\right)-x_{4}}{R_{4}} & \frac{p_{2}\left(k\right)-y_{4}}{R_{4}} & \frac{p_{3}\left(k\right)-z_{4}}{R_{4}} \end{array}\right]$$

where

(88) $$R_{i}=\sqrt{{\left(p_{1}\left(k\right)-x_{i}\right)}^{2}+{\left(p_{2}\left(k\right)-y_{i}\right)}^{2}+{\left(p_{3}\left(k\right)-z_{i}\right)}^{2}}$$

The algorithm flow of the two-level error model EKF is shown in Algorithm 1.

**Algorithm 1.** Process of two-level error model EKF.
**Algorithm 1:** Two-Level Error Model EKF**State Variables: **$x={\left[p,v,q,\delta p,\delta v,\delta q,\delta a,\delta w\right]}^{T}$**Initialization: **$x_{0},P_{0}$**Input: **$x_{k-1},P_{k-1}{,}^{1}u_{k-1},z_{k}$**Output: **${\hat{x}}_{k},{\hat{P}}_{k}$$1:\;a_{k-1}\leftarrow a_{m}^{k-1}+\delta a_{k-1}$$;\;w_{k-1}\leftarrow w_{m}^{k-1}+\delta w_{k-1}$; $u_{k-1}^{+}\leftarrow {\left[a_{k-1},w_{k-1}\right]}^{T}$$2:\;{\hat{x}}_{k}^{-}\leftarrow f_{k-1}\left(x_{k-1},u_{k-1}^{+}\right)$$3:\;P_{k}^{-}\leftarrow F_{k-1}P_{k-1}F_{k-1}^{T}+G_{k-1}{}^{2}Q_{k-1}G_{k-1}^{T}$$4:\;K_{k}\leftarrow P_{k}^{-}H_{k}^{T}{\left(H_{k}P_{k}^{-}H_{k}^{T}{+}^{3}R_{k}\right)}^{-1}$$5:\;{\hat{x}}_{k}^{+}\leftarrow {\hat{x}}_{k}^{-}+K_{k}\left[z_{k}-h_{k}\left({\hat{x}}_{k}^{-},0\right)\right]$$6:\;{\hat{P}}_{k}\leftarrow \left(I-K_{k}H_{k}\right)P_{k}^{-}$$7:\;{\hat{p}}_{k}\leftarrow {\hat{p}}_{k}^{+}+\delta {\hat{p}}_{k}^{+}$$;\;{\hat{v}}_{k}\leftarrow {\hat{v}}_{k}^{+}+\delta {\hat{v}}_{k}^{+}$$;\;{\hat{q}}_{k}\leftarrow {\hat{q}}_{k}^{+}⊗\delta {\hat{q}}_{k}^{+}$$8:\;{\hat{x}}_{k}\leftarrow {\left[{\hat{p}}_{k},{\hat{v}}_{k},{\hat{q}}_{k},\delta {\hat{p}}_{k}^{+},\delta {\hat{v}}_{k}^{+},\delta {\hat{q}}_{k}^{+},\delta {\hat{a}}_{k}^{+},\delta {\hat{w}}_{k}^{+}\right]}^{T}$ $9:\;Return:\;{\hat{x}}_{k},{\hat{P}}_{k}$

$$u_{k-1}={\left[a_{m}^{k-1},w_{m}^{k-1}\right]}^{T},\;Q_{k-1}=E\left[w_{k-1}w_{k-1}^{T}\right],\;R_{k}=E\left[r_{k}r_{k}^{T}\right].$$

## 4. Experiment Results and Discussion

In this section, we discuss the experiment results of the two-level error model EKF method based on the UWB-drone dataset [34]. We present the results in three sections. In Section 4.1, we introduce the dataset used in this paper and present the prefiltering results. In Section 4.2, the parameters in the two-level error model, which need to be acquired beforehand, are shown. Additionally, in Section 4.3, the comparison results of the two-level error model and the basic EKF method are presented.

### 4.1. Dataset Description and Prefiltering

#### 4.1.1. Dataset Description

**Figure 4:**
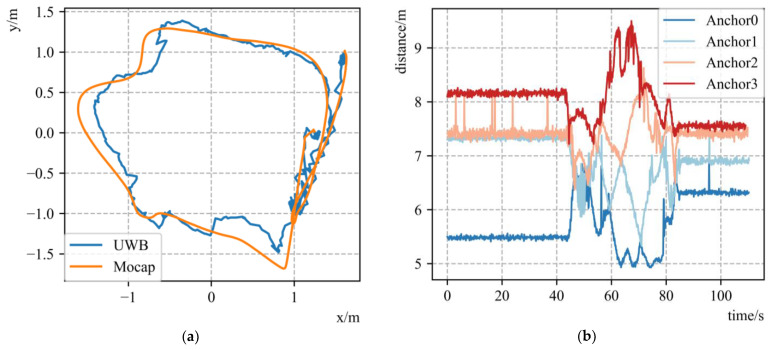
Dataset description by figures. (**a**) The x–y plane figures of UWB and Mocap positions; (**b**) the distance sequence figures of 4 anchors.

The UWB-drone dataset is about UWB-based UAV localization in GNSS-denied environments, and we selected UAV/anchor_in_room_corners to carry out the experiment. This dataset contains UAV IMU information, UWB anchor distance and position and Mocap data as the ground truth. The positions of the x–y planes in relation to UWB and Mocap are shown in Figure 4a, and the distance sequence figure of the four anchors is shown in Figure 4b.

#### 4.1.2. Data Prefiltering Results

**Figure 5:**
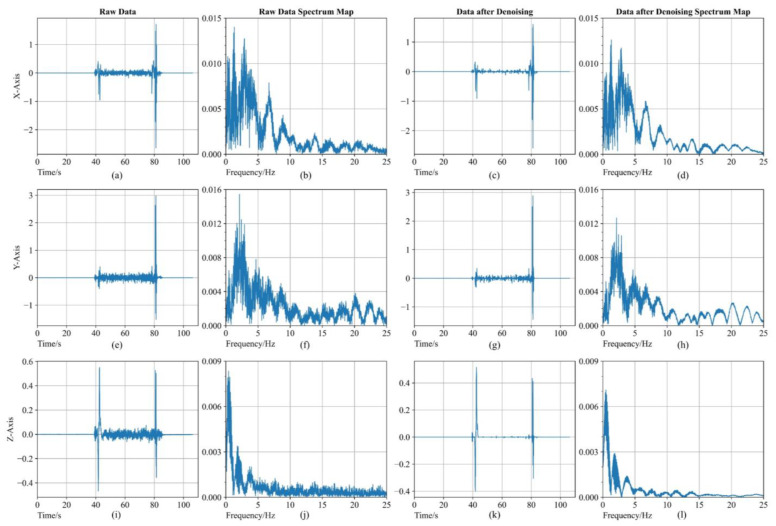
The comparison figure of gyroscope data and gyroscope data after prefiltering. (**a**,**c**,**i**) are raw data of gyroscope during UAV flight corresponding to x, y and z axes; (**b**,**f**,**j**) are raw data spectrum map of gyroscope; (**c**,**g**,**k**) are data after prefiltering corresponding to x, y and z axes; and (**d**,**h**,**l**) are spectrum map of data after prefiltering.

The data prefiltering results of the gyroscope are shown in Figure 5, with the accelerometer similarly. From the three-axes raw data figures and their spectrum maps, it can be seen that the raw signals from IMU have obvious fluctuations. After applying two-level wavelet decomposition and reconstruction from the SURE-Shrink thresholding method, the results are shown in Figure 5c,g,k, and the last column of figures corresponds to the spectrum map. The fluctuation of raw signals is significantly reduced after denoising. In the spectrum maps of data after denoising, the high-frequency signal amplitude is significantly weakened.

### 4.2. Two-Level Error Model Parameter Estimation Results

**Figure 6:**
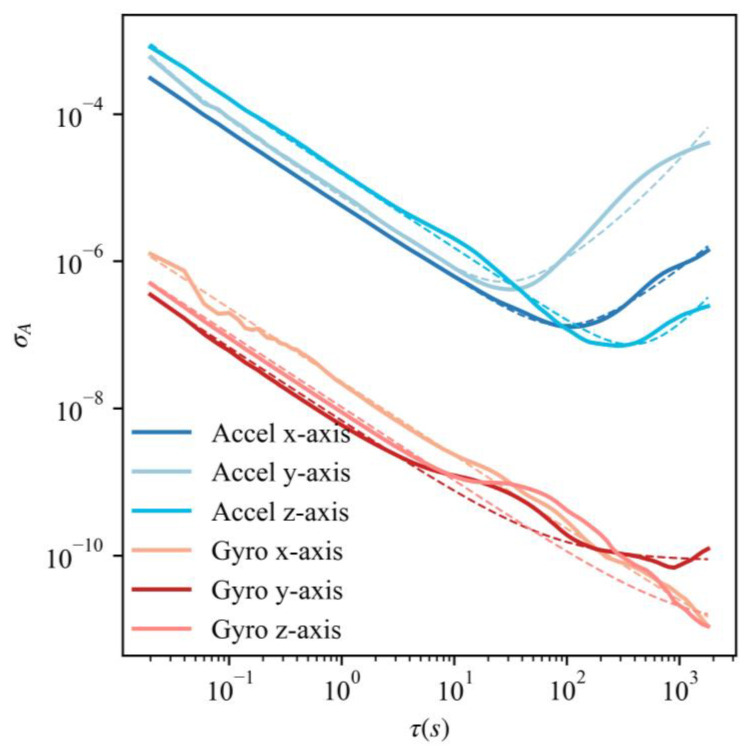
Allan variance of six-axes IMU (three-axes accelerometer and three-axes gyroscope).

In the two-level error model, especially the mechanism error model, we need to estimate the parameters $\theta =\left\{\tau_{b},\sigma_{b},\sigma_{w}\right\}$ beforehand. $\tau_{b}$ can be obtained by calculating correlation time, while $\sigma_{b},\sigma_{w}$ can be obtained from the Allan variance plot. The dataset UAV/anchor_in_room_corners does not contain long-term IMU stationary data, and the experiment was conducted on a UAV with a Pixhawk2.4 controller whose IMU was MPU6000. Thus, we performed the inertial sensor stationary experiment on MPU6000 for 5 h to obtain static IMU data, and then, we created the Allan variance plot, as shown Figure 6. In this figure, the raw data of a six-axes IMU are plotted in solid lines, and the prediction curves of six-axes data are plotted in fine-dotted lines. $\sigma_{w}$ is the strength of the rate or the acceleration white noise process and is often termed “angular/velocity random walk”, while in Figure 6, this corresponds to the curve with slope −1/2. $\sigma_{b}$ is termed “bias diffusion” and corresponds to the curve with slope 1/2. The fine-dotted curves are plotted according to the estimated parameters $\sigma_{b},\sigma_{w}$.

The parameter estimation results are shown in Table 1.

### 4.3. Two-Level Error Model-Based EKF Method Results

We now apply the two-level error model proposed in Section 3 to the UAV dataset. We show that the two-level error model can improve the attitude estimation performance of the fusion algorithm in the UAV motion process by comparing three-axes trajectory plots, which indicate attitude estimation performance, and error figures. The methods we use to compare are the EKF method based on the two-level error model, the basic EKF method and the EKF method based on the data after wavelet denoising.

**Figure 7:**
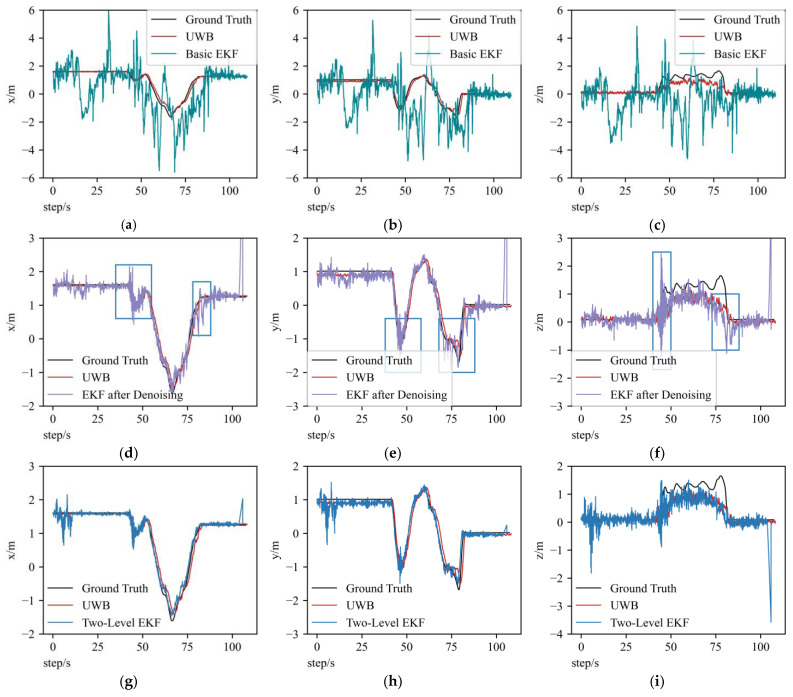
Trajectory plots of three methods (two-level error model EKF method, basic EKF method and EKF method with data after denoising) in x, y and z axes. (**g**–**i**) are trajectory estimation comparisons of two-level EKF, UWB and ground truth by Mocap. (**a**–**c**) are trajectory estimation comparisons of basic EKF method, UWB and ground truth. (**d**–**f**) are trajectory comparisons of EKF based on data after denoising, UWB and ground truth.

**Figure 8:**
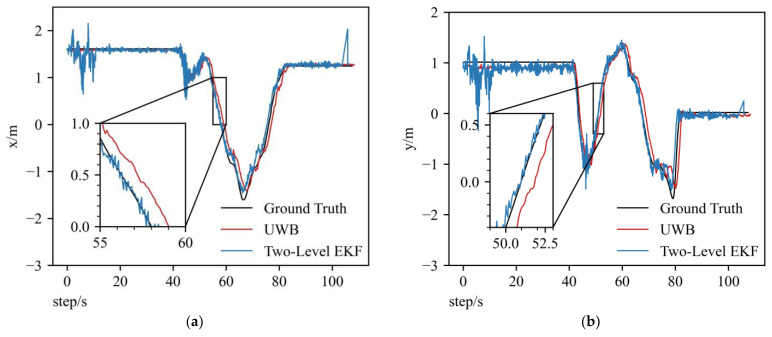
Partially enlarged trajectory plots of two-level error model EKF in x and y axes corresponding to (**a**,**b**).

Figure 7 shows the trajectory comparison of three methods in the x, y and z axes. The ground truth is plotted in black lines, and the UWB position is plotted in red lines. The sub-graphs g–i correspond to the EKF method based on the two-level error model; a–c correspond to the basic EKF method; and d–f correspond to the EKF method based on the data after denoising. By comparing d–f with a–c, we can see that the SURE-Shrink wavelet denoising method brings a large performance enhancement to the EKF fusion method, and the mean positioning error is eliminated to 0.457 m from 1.949 m, as shown in Figure 8. At the points where the UAV motion state changes, applying the two-level error model can provide better computational stability, which manifests by less curve fluctuations in the figures—blue widow parts in d–f as examples.

**Figure 9:**
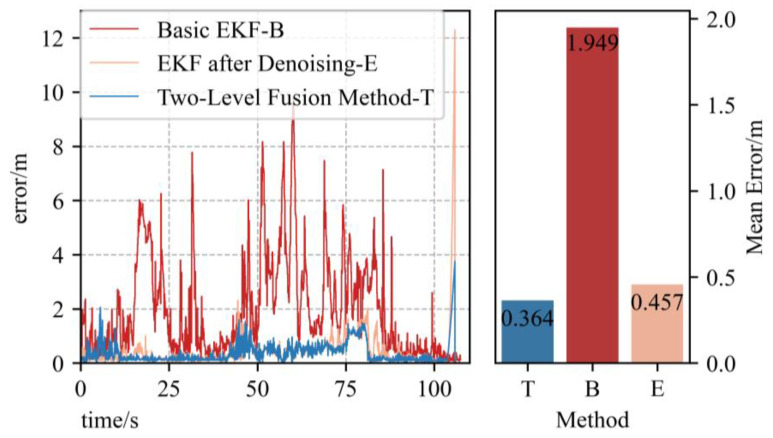
Error figure of all three methods, basic EKF (“B” for short), EKF based on data after denoising (“E” for short) and two-level error model EKF method (“T” for short). The mean errors are on the right.

Applying the EKF fusion method based on raw IMU data does not effectively improve the UAV positioning accuracy, even though the UWB positions are quite close to the ground truth, as shown in Figure 7a–c. However, by applying the two-level error model, the fusion positions are closer to ground truth than the UWB positions, as shown partially enlarged in Figure 9. At the beginning of EKF based on the two-level error model fusion, the curve has large fluctuations because the initial gain matrix deviates far from the optimal value. Compared to x and y axes’ results of the two-level error model EKF method, the z-axis result has larger fluctuations. The reasons that cause these fluctuations can be divided into two aspects: one is that the UWB positioning accuracy is poorer in the z-axis than the x and y axes, which causes large fluctuations in the UWB position results; the other is that the inertial data in this UAV dataset generally contain much vibration, and these two reasons explain the large fluctuations.

Figure 9 shows the error figures of the three methods. Applying the EKF fusion method on raw inertial data causes a large error, and the average error of the basic EKF method is 1.949 m. After applying the SURE-Shrink wavelet denoising method, the fusion accuracy is greatly improved, and the average error is eliminated to 0.457 m; the accuracy is improved by 76.6%. After applying the two-level error model, the computational stability is largely enhanced at UAV motion state change points, and the accuracy is improved by 84.3%.

## 5. Conclusions

This paper set out to modify the INS-based fusion positioning method by providing generic noise models. In this paper, the IMU wavelet denoising method based on SURE-Shrink threshold was summarized, and the two-level error model was proposed, which includes the mechanism error model and the propagation error model. The mechanism error model was established from stochastic process theory, and the propagation error model was derived from navigation principles; both compensate for the inertial sensor errors. We derived the EKF fusion method based on the two-level error model, and the experimental verification was carried out with UWB measurements. The experimental results suggest that applying the wavelet denoising method could largely improve positioning accuracy by 76.6% compared to the basic EKF method. Additionally, applying the two-level error model could further improve positioning accuracy by 84.3% compared to basic EKF. Meanwhile, at the points where the UAV motion state changes, using the two-level error model could provide higher computational stability and less trajectory curve fluctuations.

At the end of the curve for the z-axis in the two-level error model EKF method in Figure 7i, huge data fluctuations exist in the fusion. This is an interesting phenomenon, and we do not consider the case of unknown inputs in the system. This is an issue for future research to explore.

## Figures and Tables

**Table 1:** Results of model parameter estimation of MPU6000.

Sensor	Axis	$\sigma_{w}$	$\sigma_{b}$
Accelerometer	x	$2.419\times 10^{-3}$	$4.402\times 10^{-5}$
	y	$2.725\times 10^{-3}$	$1.63\times 10^{-4}$
	z	$3.9\times 10^{-3}$	$1.403\times 10^{-5}$
Gyroscope	x	$1.52\times 10^{-4}$	0
	y	$8.2\times 10^{-5}$	0
	z	$1.02\times 10^{-4}$	0

## Data Availability

Not applicable.

## Appendix A

(89)
$$\begin{array}{l} F_{11}=I_{3\times 3}\\ F_{12}=I_{3\times 3}\Delta t\\ F_{22}=I_{3\times 3}\\ F_{27}=C^{k-1}\Delta t\\ F_{44}=I_{3\times 3}\\ F_{45}=I_{3\times 3}\Delta t\\ F_{55}=I_{3\times 3}\\ F_{57}=C^{k-1}\Delta t\\ F_{66}=\Omega \left(w_{m}^{k-1}\right)\\ F_{68}=Q\left(q_{m}^{k-1}\right)\\ F_{77}=I_{3\times 3}-\frac{\Delta t}{\tau_{b,a}}\\ F_{78}=I_{3\times 3}-\frac{\Delta t}{\tau_{b,w}} \end{array}$$
